# Alumni Matters

**Published:** 1881-08

**Authors:** 


### ALUMNI MATTERS.

The following circular, from the duly appointed committee to the Alumni of the Ohio College of Dental Surgery, is interesting and should receive the prompt attention of every one to whom it is sent. Every Alumnus should have a pride in his *alma mater*, and do whatever he could to promote her true interest and effort to impart thorough professional education:

CINCINNATI, April 5, 1881.

DEAR DOCTOR: At a meeting of the Alumni Association of the Ohio College of Dental Surgery, held March 3d, 1881, a committee, consisting of five members of the Association, was appointed to prepare for publication a history of the college and of each alumnus, and such other matters as would be of interest to the Association.

The committee desire a complete history of the college from its organization; as complete a history of each alumnus as possible, and biographical sketches of deceased alumni.

To this end the committee invite your attention to the enclosed list of questions, to which it is earnestly hoped you will take no exceptions and give a few minutes consideration at your earliest conveniences and forward to the secretary, in order that the report may be as complete as possible.

N. S. HOFF, D. D. S.

GEO. WATT, M. D., D. D. S,

W. D. KEMPTON, M. D., D. D. S.

C. I. KEELY, D. D. S.

E. G. BETTY, D. D. S., Secretary.

126 W. Ninth St. Cincinnati, O.

Name in full. Place and date of birth. What, if any, literary institutions did you attend? Where, when and with whom did you begin the study of dentistry? What other, if any, dental colleges did you attend? State the localities in which you have practiced, and your present location (give the time in each locality. Are you a graduate of medicine? (state where and when you graduated). Of what dental societies have you been a member? What positions of honor have you held in or out of the profession? What have you contributed to dental literature? Married or single? Can you give any facts concerning any deceased member of your class? Give any facts of interest concerning the college, its faculty or alumni, in your possession, not contained in the above questions.

